# Correction: Complete chloroplast genomes of *Asparagus aethiopicus* L., A. *densiflorus* (Kunth) Jessop ‘Myers’, and A. *cochinchinensis* (Lour.) Merr.: Comparative and phylogenetic analysis with congenerics

**DOI:** 10.1371/journal.pone.0303739

**Published:** 2024-05-09

**Authors:** The PLOS ONE Staff

## Notice of republication

This article was republished on April 30th, 2024, to correct an error in the level of headings that were introduced during the typesetting process: the heading Medicinal Application was erroneously displayed as a main level heading instead of as a subsection of the Introduction. The publisher apologizes for the error. Please download this article again to view the correct version. The originally published, uncorrected article and the republished, corrected articles are provided here for reference.

## Supporting information

S1 File Originally published, uncorrected article. (PDF)

S2 File Republished, corrected article. (PDF)
