# Supplementary material for: Complete chloroplast genomes of Asparagus aethiopicus L., A. densiflorus (Kunth) Jessop ‘Myers’, and A. cochinchinensis (Lour.) Merr.: Comparative and phylogenetic analysis with congenerics
Source: PLoS One. 2022 Apr 25;17(4):e0266376. doi: 10.1371/journal.pone.0266376 (PMC9037925; doi:10.1371/journal.pone.0266376)
Supplement: S1 Fig — (PDF) [file pone.0266376.s001.pdf]

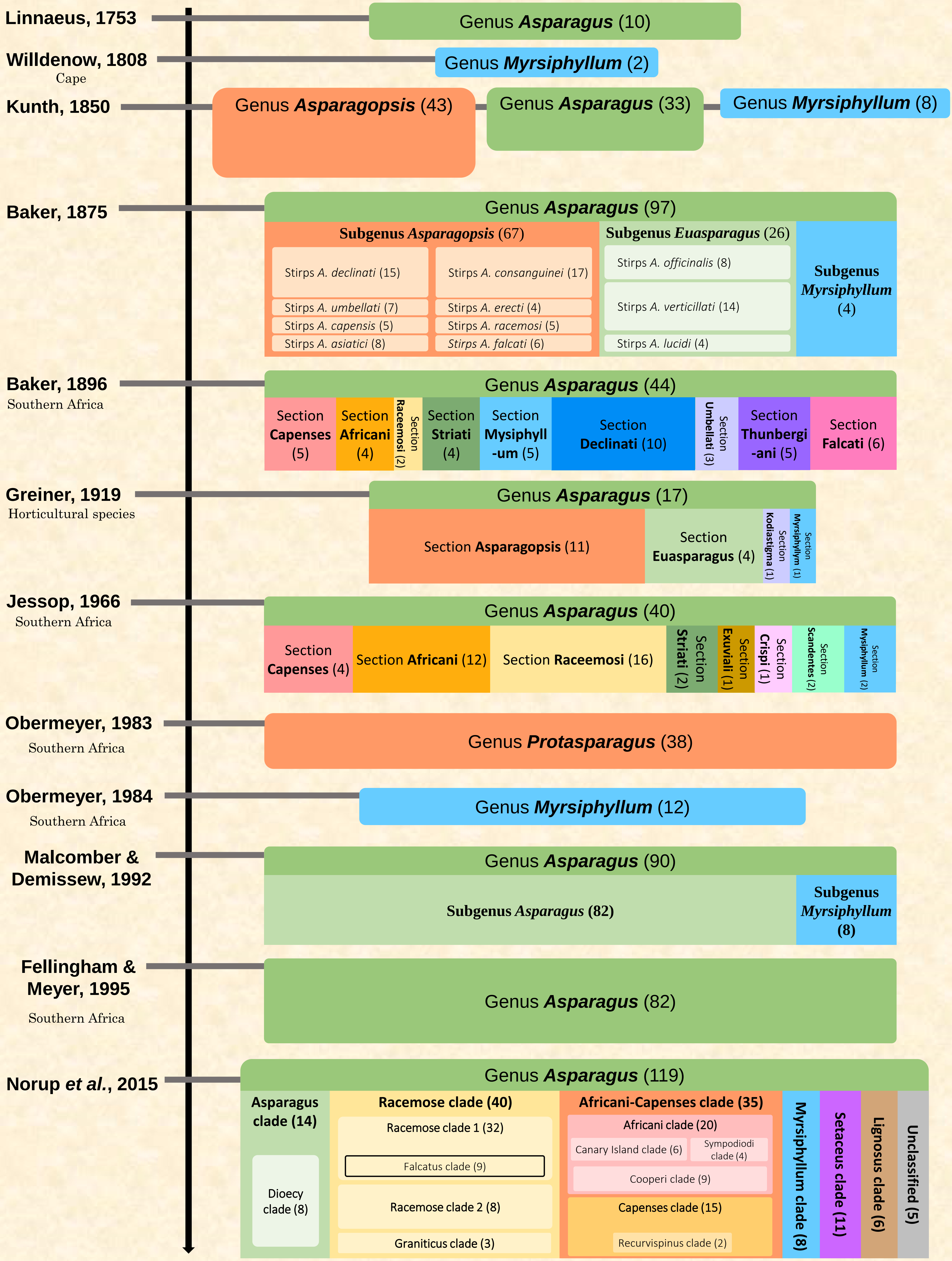

S1 Figure. The historical changes on the generic subdivision of the genus *Asparagus*

Remark: This infographic was not drawn proportionally. Numbers in the brackets indicated the number of species classified into such categories based on the publications of specified author(s). Specified regions or category of plants were indicated below the author(s) and publication years.

Reference

(ordered by year)

Linnaei, C. (1753). *Asparagus*. In *Species Plantarum* (Vol. 1, pp. 313-314). Imprensis Laurentii Salvii. Retrieved 15th August 2021, from: https://doi.org/10.5962/bhl.title.669

Willdenow, C. L. (1808). Nähere Bestimmung einiger Liliengewächse, besonders aber derjenigen, die zur sechsten Klasse und dritten Ordnung des Linneschen Systems gehören. In *Der Gesellschaft Naturforschender Freunde zu Berlin Magazin für die neuesten Entdeckungen in der gesammten Naturkunde* (pp. 14-29). Realschulbuchhandlung. Retrieved 11th August 2021, from: http://resolver.sub.uni-goettingen.de/purl?PPN608227714_0002

Kunth, C. S. (1850). II. Asparageae Kunth. In *Enumeratio plantarum* (Vol. 5, pp. 57-110). Stutgardiae et Tubingae. Retrieved 11th August 2021, from: https://bibdigital.rjb.csic.es/records/item/11045-redirection

Baker, J. G. (1875). Revision of the Genera and Species of Asparagaceae. The Journal of the Linnean Society, Botany, 508-632. Retrieved 10th August 2021, from: https://www.biodiversitylibrary.org/item/8365

Baker, J. G. (1896). II. *Asparagus*, Linn. In H. H. William & S. O. Wilhelm (Eds.), *Flora capensis: being a systematic description of the plants of the Cape colony, Caffraria, & Port Natal* (Vol. 1, pp. 256-274). Hodges, Smith, and Co. Retrieved 11th August 2021, from: https://doi.org/10.5962/bhl.title.821

Greiner, T. (1919). *Asparagus*. In L. H. Bailey (Eds.), *The Standard Cyclopedia of Horticulture* (1st ed., pp. 406-411). The Macmillan Company. Retrieved 12th August 2021, from: https://doi.org/10.5962/bhl.title.23351

Jessop, J. P. (1966). The Genus *Asparagus* in South Africa. Bothalia, 9 (1), 31-96. Retrieved 25th May 2021, from: https://www.jstor.org/stable/41761647

Obermeyer, A. A. (1983). *Protasparagus* Oberm. nom. nov.: new combinations. South African Journal of Botany, 2(3), 243-244. Retrieved 4th June 2021, from: https://cyberleninka.org/article/n/676191/viewer

Obermeyer, A. A. (1984). Revision of the genus *Myrsiphyllum* Willd. Bothalia 15 (1 & 2), 77 – 88. Retrieved 4th June 2021, from: https://journals.abcjournal.aosis.co.za/index.php/abc/article/view/1106

Malcomber, S. T. & Demissew, S. (1993). The Status of *Protasparagus* and *Myrsiphyllum* in the Asparagaceae. Kew Bulletin, 48 (1), 63-78. Retrieved 4th June 2021, from: https://doi.org/10.2307/4115749

Fellingham, A. C. & Meyer, N. L. (1995). New combinations and a complete list of *Asparagus* species in southern Africa (Asparagaceae). Bothalia, 25 (2), 205-209. Retrieved 30th July 2021, from: https://www.researchgate.net/publication/283987189_New_combinations_and_a_complete_list_of_Asparagus_species_in_southern_Africa_Asparagaceae

Norup, M. F, Petersen, G., Burrows, S., Bouchenak-Khelladi, Y., Leebens-Mack, J., Pires, J. C., Linder, H. P. & Seberg, O. (2015). Evolution of *Asparagus* L. (Asparagaceae): Out-of-South-Africa and multiple origins of sexual dimorphism. Molecular Phylogenetics and Evolution, 92, 25-44. Retrieved 8th June 2021, from: http://doi.org/10.1016/j.ympev.2015.06.002
