# Supplementary material for: Complete chloroplast genomes of Asparagus aethiopicus L., A. densiflorus (Kunth) Jessop ‘Myers’, and A. cochinchinensis (Lour.) Merr.: Comparative and phylogenetic analysis with congenerics
Source: PLoS One. 2022 Apr 25;17(4):e0266376. doi: 10.1371/journal.pone.0266376 (PMC9037925; doi:10.1371/journal.pone.0266376)

# S3 Figure. Visualisation of the alignments of 7 *Asparagus* chloroplast genomes using *Asparagus aethiopicus* L. as a reference

**Page 1 – from 1 bp to 53000 bp**

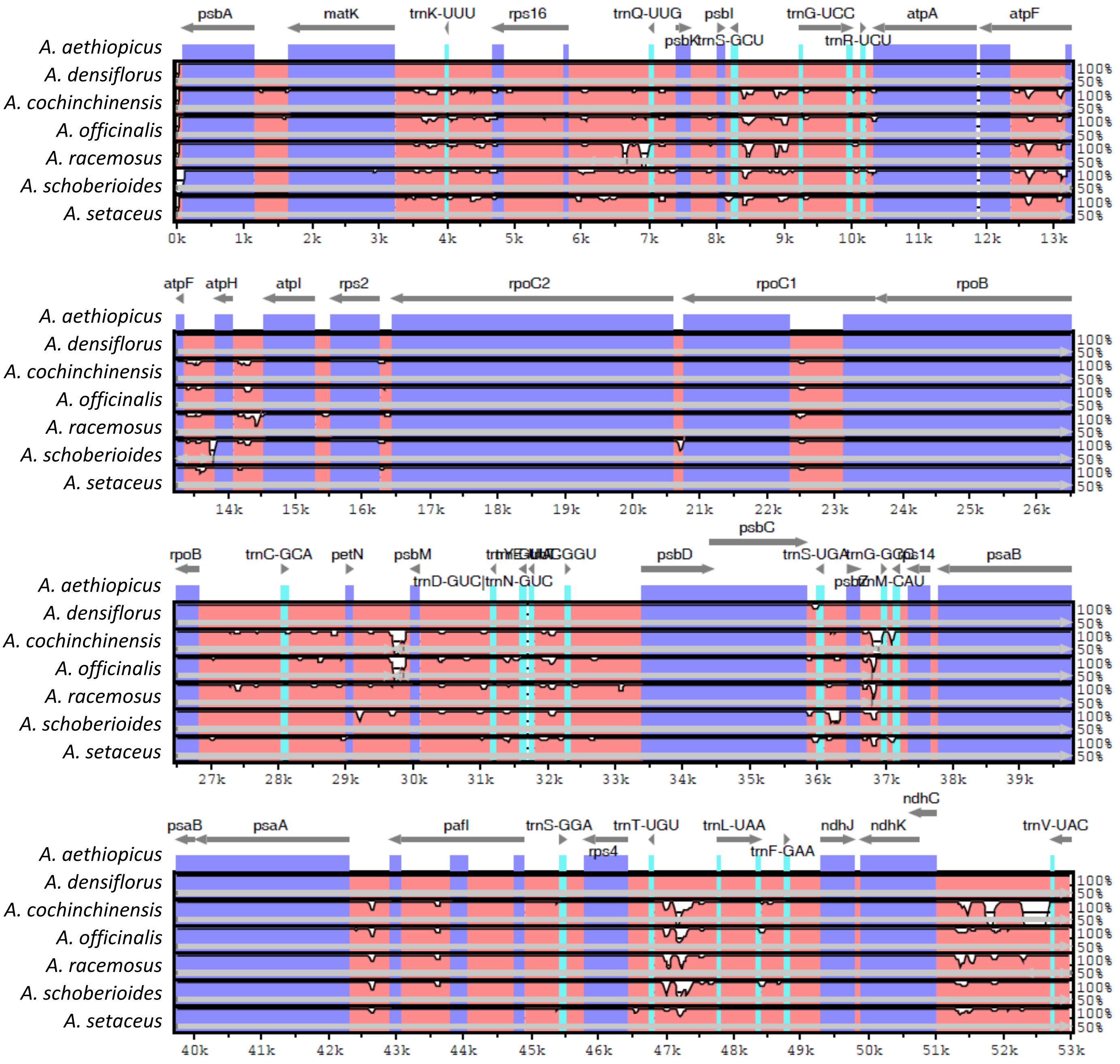


**Legend:** The vertical scale indicates the percentage of identity, ranging from 50 to 100%. The horizontal axis indicates the coordinates within the chloroplast genome. Protein-coding regions are labeled with blue while the conserved non-coding sequences (CNS) are labeled with red.

**Key**

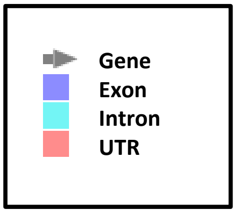

# S3 Figure. Visualisation of the alignments of 7 *Asparagus* chloroplast genomes using *Asparagus aethiopicus* L. as a reference

**Page 2 – from 53000 bp -105999bp**

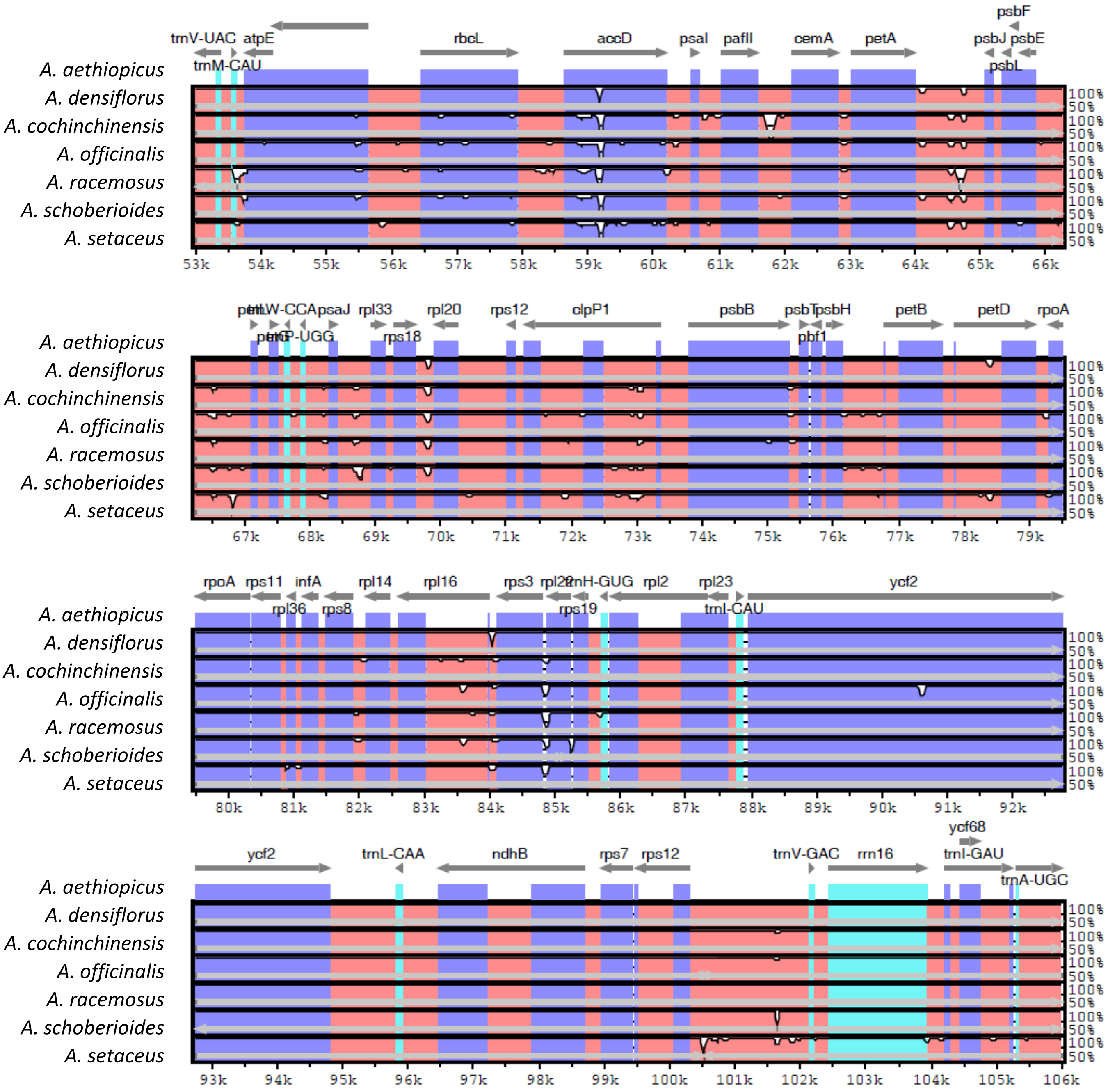


**Legend:** The vertical scale indicates the percentage of identity, ranging from 50 to 100%. The horizontal axis indicates the coordinates within the chloroplast genome. Protein-coding regions are labeled with blue while the conserved non-coding sequences (CNS) are labeled with red.

**Key**

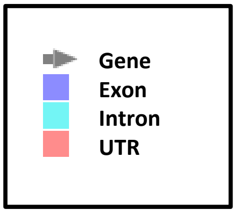

# S3 Figure. Visualisation of the alignments of 7 *Asparagus* chloroplast genomes using *Asparagus aethiopicus* L. as a reference

**Page 3 – from 105917 bp -157068 bp**

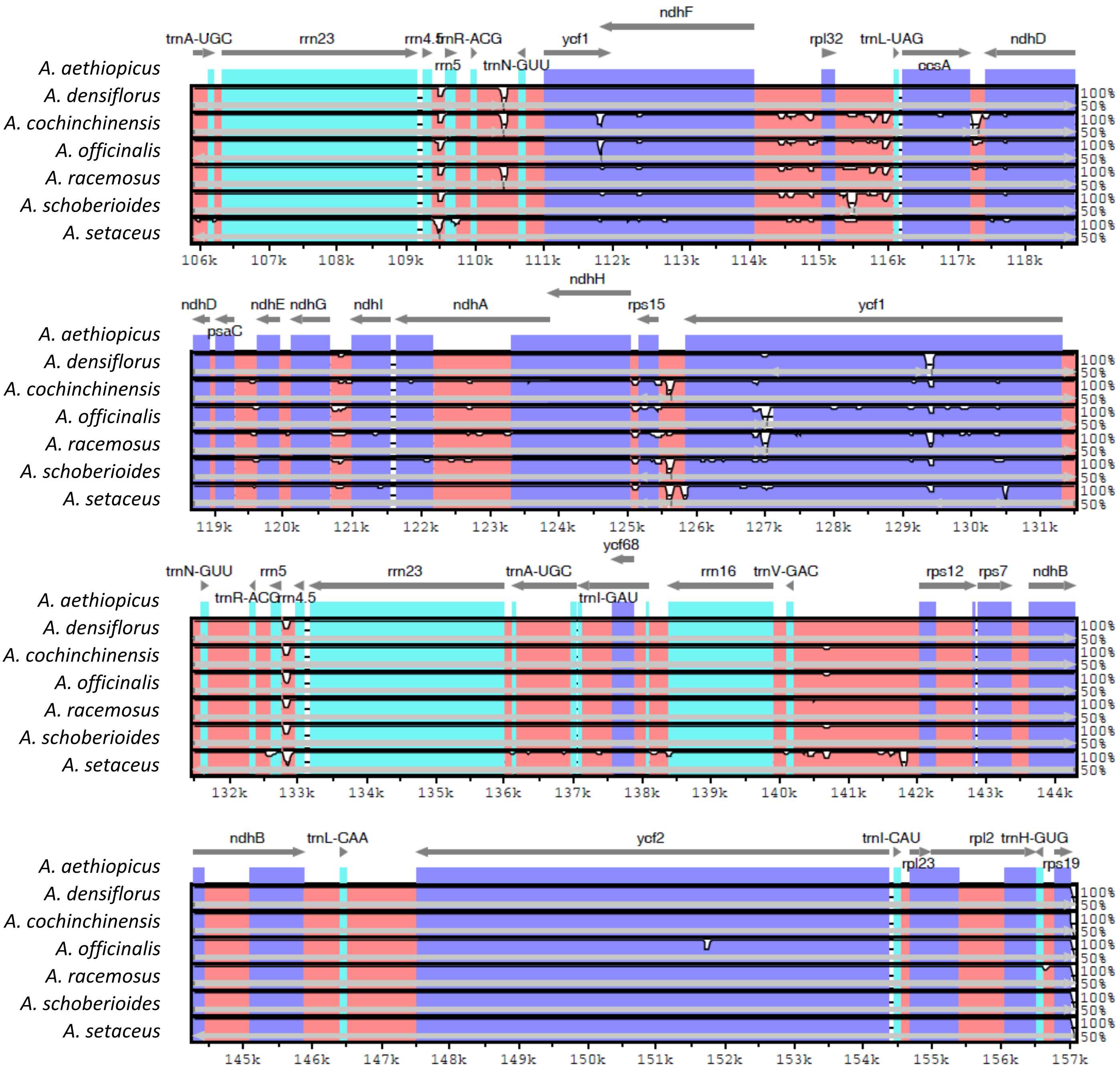


**Legend:** The vertical scale indicates the percentage of identity, ranging from 50 to 100%. The horizontal axis indicates the coordinates within the chloroplast genome. Protein-coding regions are labeled with blue while the conserved non-coding sequences (CNS) are labeled with red.

**Key**

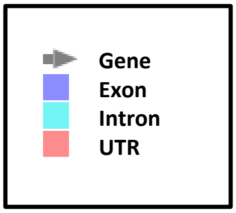

Supplement: S2 Fig — (PDF) [file pone.0266376.s002.pdf]
