# Supplementary material for: Complete chloroplast genomes of Asparagus aethiopicus L., A. densiflorus (Kunth) Jessop ‘Myers’, and A. cochinchinensis (Lour.) Merr.: Comparative and phylogenetic analysis with congenerics
Source: PLoS One. 2022 Apr 25;17(4):e0266376. doi: 10.1371/journal.pone.0266376 (PMC9037925; doi:10.1371/journal.pone.0266376)
Supplement: S3 Fig — (PDF) [file pone.0266376.s003.pdf]

0 1 2 3 4 5 6 7 8 9 10
cm
copyright reserved
胡秀英植物標本館
SHIU-YING HU HERBARIUM

Shiu-Ying Hu Herbarium, CUHK

Plants of Hong Kong

LILIACEAE

Asparagus densiflorus (Kunth) Jessop 'Myersii' 狐尾天門冬

CUHK

Herbs growing on a concrete container with a *Bougainvillea spectabilis* Willd. outside Run Run Shaw Hall. Cladodes green. Flowers white. Mature fruits red. Collected for DNA Lab (Asparagaceae Project).

Coll. K.H. Wong 092 CUSLSH2773 26 Oct 2020

Det. K.H. Wong

CUHK05890

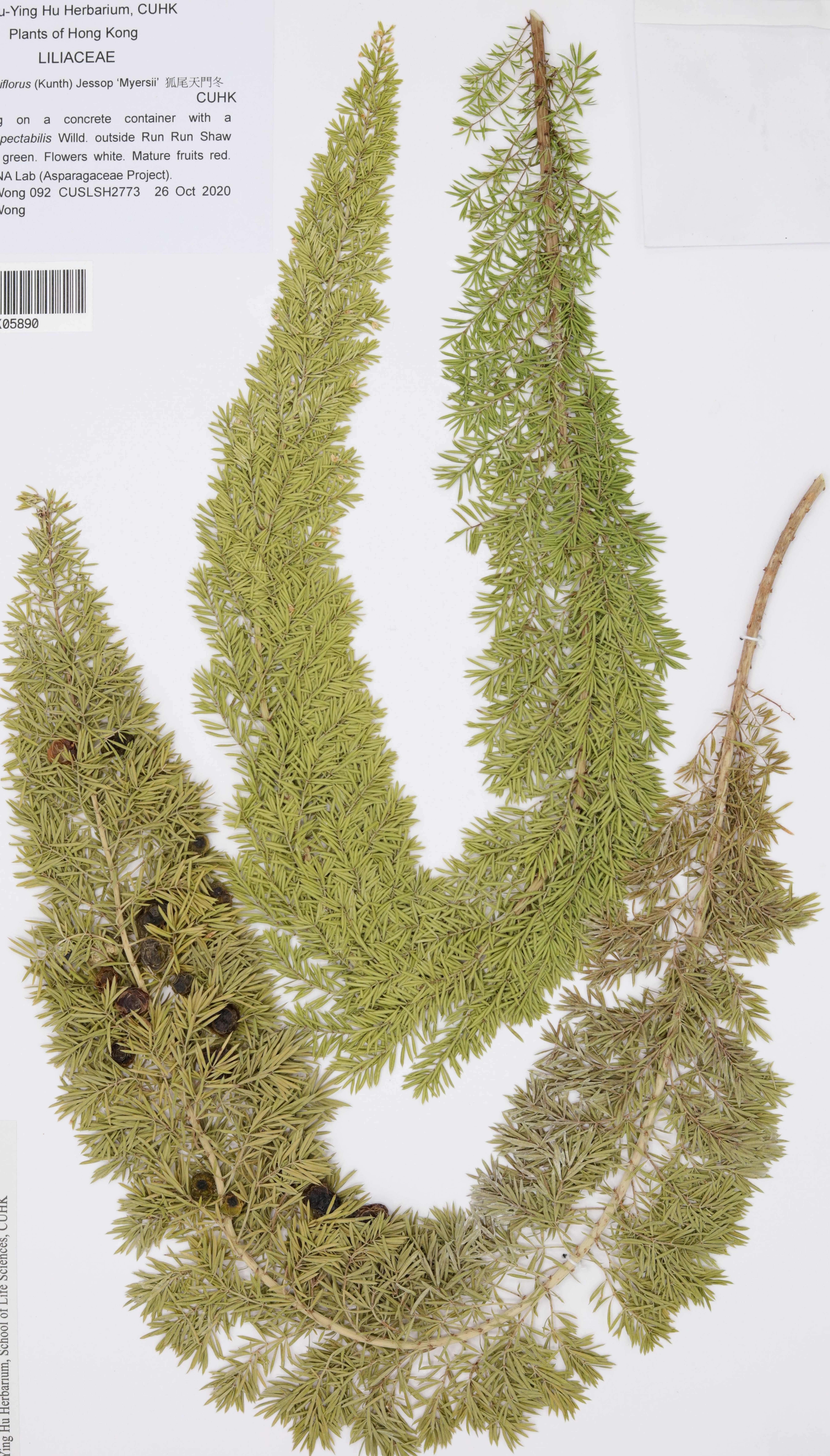

Asparagus densiflorus (Kunth) Jessop 'Myers'

Det. K.H. Wong Date: 8th June, 2021

Shiu-Ying Hu Herbarium, School of Life Sciences, CUHK

0 1 2 3 4 5 6 7 8 9 10
cm
copyright reserved

胡秀英植物標本館
SHIU-YING HU HERBARIUM

CUHK05891

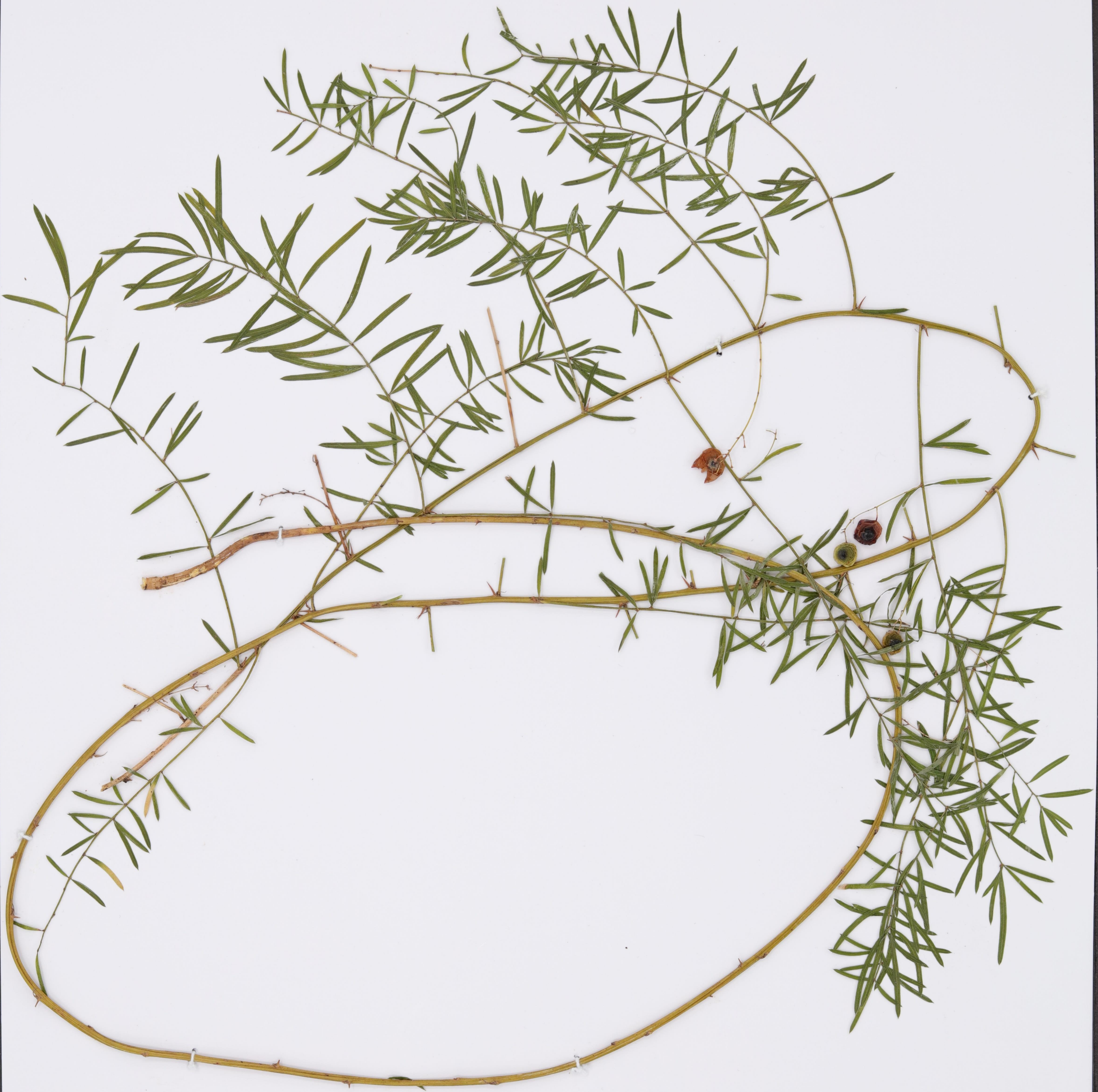

Shiu-Ying Hu Herbarium, CUHK

Plants of Hong Kong

LILIACEAE

Asparagus densiflorus (Kunth) Jessop 'Sprengeri'

非洲天門冬

CUHK

Herb grows under the shade of a ficus tree near New Asia Amphitheatre. Base of stem very prickled. Cladodes green. Fruits light green when young, red when mature. Same population as K.H. Wong 110. Collected for DNA Lab (Asparagaceae Project).

Coll. K.H. Wong 109 CUSLSH2801 05 Nov 2020

Det. K.H. Wong

Asparagus aethiopicus L. var. *aethiopicus* 武竹

Det. K. H. Wong Date: 11th June, 2021

Shiu-Ying Hu Herbarium, School of Life Sciences, CUHK

0 1 2 3 4 5 6 7 8 9 10
cm
copyright reserved
胡秀英植物標本館
SHIU-YING HU HERBARIUM

CUHK05892

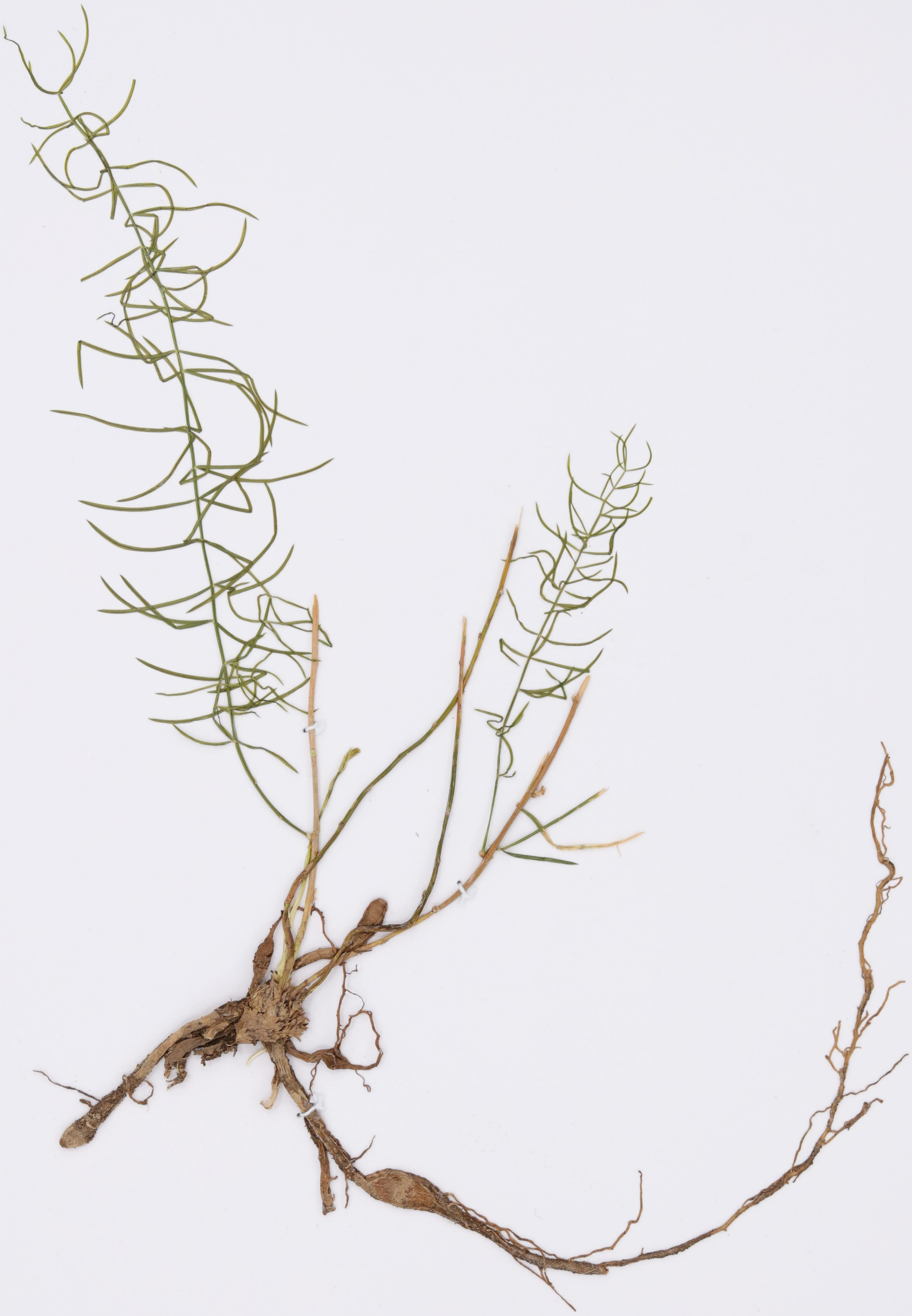

Shiu-Ying Hu Herbarium, CUHK

Plants of Hong Kong

LILIACEAE

Asparagus cochinchinensis (Lour.) Merr. 天門冬

CUHK

Herbs growing on the edge of concreted slope next to Fok Ying Tung Remote Sensing Science Building. Cladodes green. Scale-like leaves whitish. Root tubers and fibrous roots brownish. In the same population as K.H. Wong 091 & 108. Collected for DNA Lab (Asparagaceae Project).

Coll. K.H. Wong 107 CUSLSH2799 05 Nov 2020

Det. K.H. Wong
