# Supplementary material for: Complete chloroplast genomes of Asparagus aethiopicus L., A. densiflorus (Kunth) Jessop ‘Myers’, and A. cochinchinensis (Lour.) Merr.: Comparative and phylogenetic analysis with congenerics
Source: PLoS One. 2022 Apr 25;17(4):e0266376. doi: 10.1371/journal.pone.0266376 (PMC9037925; doi:10.1371/journal.pone.0266376)
Supplement: Attachment — Submitted filename: Rebuttal Letter.docx [file pone.0266376.s006.docx]

Dear Dr. Šiler, Reviewer 1 and Dr. Sheng,

**Rebuttal Letter**

I am sincerely writing to you to response all your comments in the Decision Letter in 18^th^ November 2021. Our replies are listed as below.

Comment 1. As suggested by Reviewer #1, literature update is highly recommended, as the authors did not present the latest findings related to the scientific matter. Moreover, an in depth authors' elaboration of the present text regarding the literature update is expected.

**Reply:** Agreed. We have added the relevant articles in the reference list and elaborated their findings in the sections of introduction, result and discussion.

Comment 2. The whole text would hugely benefit if proofread by a native English speaker or a professional editing agency…

**Reply:** Agreed. We will employ a professional editing agency to proofread the article after all parties agreed with the content.

Comment 3. L36: "species" should not stand italicized.

**Reply:** Agreed and revised.

Comment 4. L39: "Asparagus have been widely applied..." - a colloquial expression. Suggestion: "Many species belonging to the Asparagus genus have been widely applied..."

**Reply:** Agreed. Amendment was made as follows:

“Many species belonging to the genus *Asparagus* have been widely applied in different aspects of human society”.

The genus name was italicized and put after the word “genus” according to the rule of ICN.

Comment 5. L41, L58 and elsewhere in the text: "anthropocentric usage of Asparagus", "while some Asparagus were used"... - the same comment as the previous.

**Reply:** Agreed and amended as follows:

L60-61: Adapting to drought conditions, ***Asparagus* species** have evolved characteristic morphology.

L71-72: Below we briefly discussed the anthropocentric usage of **some *Asparagus* species**…

L89: …while **some *Asparagus* species** were used as charm to increase fertility…

Comment 6. L47 and further in the text: Please use "A." to abbreviate the genus name when introducing a species name.

**Reply:** We agreed with the guidelines of PLOS One. We had adopted abbreviated genus name (*A.*) for most of the binominal names in the article. However, we would like to keep the presentation of full scientific name for the sentences below, in order to (i) obey the rules of ICN/ICNCP, (ii) accurately present the nomenclatural treatment, and (iii) present the original record of literatures.

L165-167: In 1890, Regel published the name ***Asparagus sprengeri*** based on the cultivated plants growing in Natal

L168-169: The name ***Asparagus sprengeri* Regel** hence is the scientific name of sprengeri asparagus. [Remarks: the species epithets “***sprengeri***” is amended in lower case instead of “*Sprengeri*” in the first submission]

L171-172: Jessop synonymized *A. sprengeri* Regel under the new combination ***Asparagus densiflorus* (Kunth) Jessop** based on morphology and geographical distribution

L178-179: In 1767, Linnaeus published the name ***Asparagus aethiopicus* L.** in the *Species Plantarum*

L187-188: Both names ***Aspararagopsis aethiopica*** (and later, ***Asparagus aethiopicus***) and ***Asparagopsis densiflora*** were adopted in parallel for 116 years, from 1850 to 1965.

L197-200: Straley and Utech also adopted the scientific name *A. aethiopicus* for sprengeri asparagus, and stated “***Asparagus densiflorus* (Kunth) Jessop** has been misapplied to this species” in *Flora of North America North of Mexico* (2004)

L207-210: …in terms of its habitats, growing habits and reproductive characters, fits the circumscription of ***Asparagus aethiopicus* L.** in the monograph. Therefore, we adopt the scientific name ***Asparagus aethiopicus* L.** for sprengeri asparagus throughout this study.

L218-220: The first binomial name of foxtail asparagus, ***Asparagus myersii***, was raised anonymously in an unknown time, while the name ***Asparagopsis densiflora*** was validly published in 1850 by Kunth (S2 Table)

L222-223: In 1966, Jessop mentioned the name ***Asparagus myersii* Hort.** “had never been validly published”

L239-241: …, we follow the treatment of some taxonomists and scientists [1,4-5,11,51,64,76,83], adopting the scientific name ***Asparagus densiflorus* Jessop (Kunth) ‘Myers’** for foxtail asparagus throughout this study.

L306: Remains the full name of *Asparagus aethiopicus* L., *Asparagus densiflorus* (Kunth) Jessop 'Myers' and *Asparagus cochinchinensis* (Lour.) Merr. were used in **Table 1** which indicates the authentication of specimens.

L614-616: We agreed with Batchelor & Scott (2006) that foxtail asparagus should be a cultivar of *A. densiflorus* [67], and hence the legitimate name should be ***Asparagus densiflorus* (Kunth) Jessop ‘Myers’**.

L647-648: One is to combine only the genus name with the cultivar epithet, as ***Asparagus* ‘Myers’**…

L649-651: Another treatment is the combination of the common name and the cultivar epithet, as **Asparagus ‘Myers’**… [In this case “Asparagus” is not italic and not a genus name]

Comment 7. L77 and elsewhere in the text: Do not capitalize common plant names such as "Garden Asparagus". Write "garden asparagus".

**Reply:** Agreed. All are amended.

**Comments from Reviewer 1:**

The Authors have carried out a study based on the sequencing of the Complete Chloroplast Genome of three species (A. aethiopicus, A. densiflorus ‘Myers’ and A. cochinchinensis) belonging to the Asparagus genus. The results obtained were assembled with chloroplast genomes of other four Asparagus species (A. setaceus, A. racemosus, A. schoberioides Asparagus officinalis L.) on NCBI. The manuscript could bring a significative impact for further studies aimed to widen the knowledge of the Asparagus genus and the phylogenetic relationships among Asparagus spp. In my opinion, the paper could be published after a few modifications that might improve this manuscript.

The main criticism to this paper is that I have lacked the mention of some studies employing cpDNA that have been previously developed in the Asparagus genus ( Lee et al 1996; Lee et al 1997; Kanno et al 1997; Seng et al 2017 and Li et al 2019). I was impressed about the comprehensive and detailed revision on the taxonomic classifications in the asparagus genus that have been published since the eighteen century to date. However, previous studies employing cpDNA of different Asparagus spp. are not mentioned in the manuscript. In my opinion these studies should be mentioned in the Introduction section of the manuscript. I think that at least the studies developed by Lee et al. (1997) and Seng et al. (2017) are recommended to be also used in the discussion of the manuscript because the findings of the present study can be compared to a certain extent with the obtained by these two studies.

**Reply:** Thank you so much for your appreciation! We agreed with your suggestions and advices. The suggested literatures were already included in the sections of introduction, result and discussion with appropriate elaboration.

Other comments and suggestions for the Authors:

> Abstract: The authors wrote: “Conducting comparative and phylogenetic analysis with congeneric species, four cpDNA on NCBI were included in this study”. The scientific name of these four species **(A. setaceus, A. racemosus, A. schoberioides Asparagus officinalis L.**) they should be included in this section of the manuscript.

**Reply:** Agreed. The scientific names of the four species were included accordingly.

> lines 35-37: In these lines is written the following sentence: “These aforementioned characteristics were evolved by Asparagus species to adapt to arid environment”. I think that this sentence must be support by a cite/s

**Reply:** Agreed. The reference source (Dahlgren *et al.*, 1985 and Judd, 2001) were cited accordingly.

> lines 43-44: It is written: “of the two studied Asparagus species were discussed in detail” Is it correct? Two (A. aethiopicus, A. densiflorus ‘Myers’) or three species (A. aethiopicus, A. densiflorus ‘Myers’, A. cochinchinensis)?
**Reply:** Agreed. The word “two” was deleted from this sentence.

> Lines 78- 79. The authors wrote: “However, the gene pool of A. officinalis is relatively limited” In my opinion there are other studies such as Geoffriau et al 1992, Moreno et al 2006 or Mercati et al 2015 that must be cited instead of the study carried out by Stajner et al 2002

**Reply:** Agreed. We cited your suggested source of literature instead of the one of Stajner *et al*., 2002.

> lines 79-80: I suggest modifying the following sentence: “The species is susceptible to multiple diseases” by the species is susceptible to multiple biotic and abiotic stresses…
**Reply:** Agreed. Amendment was done.

**Comments from Reviewer 2:**

The introduction part is chaotic in this manuscript, which shoule be rewritten；Please pay attention to some mistakes in your grammar and spelling, and I have labelled these shortcomings in this paper; the content is complete, and the result is clear; and I suggest this paper should be accepted after minor revison.

**Reply:** Thank you for your comments. We have rewritten the introduction. For the grammar and spelling, we have revised it accordingly, and will sent the manuscript to a professional agency for further proofread after all parties agreed with the content.

Other comments and suggestions for the Authors:

Line 72. Hong Kongwhere

**Reply:** Agreed and amended. Space between “Kong” and “where” was added.

Line 233. S2 Table

**Reply:** The sentence in Line 233 had been deleted in the latest version. The usage of S2 Table in other parts of the manuscript were put in brackets.

Line 354. The pseudogene ycf1...

**Reply:** Agreed. The gene name (*ycf1*) was italicized.

Line 471. ranging from 5624 to 5460 bp ...

**Reply:** Agreed. The unit was given for each number as “5624 bp to 5460 bp”.

Line 481. *trnS-trnG* (Pi = 0.17)

**Reply:** Agreed. The sentence was revised as “Among these hotspots, *accD-psaI* was the most variable region (Pi=0.023), followed by *ccsA* (Pi=0.020) **and then** *trnS-trnG* (Pi = 0.17).”

Line 549. [4,Error! Reference source not found.,65,74].

**Reply:** Agreed. The reference source (Straley & Utech, 2003) was updated.

Line 604. The second group consisting…

**Reply:** Agreed. The sentence was revised as "The second group **consisting of**".

Line 628. S4 Table

**Reply:** Sorry. We have no idea of this amendment. Please further advice.

**Comments from the Academic Editor:**

1. Please ensure that your manuscript meets PLOS ONE's style requirements, including those for file naming. The PLOS ONE style templates can be found at 
<https://journals.plos.org/plosone/s/file?id=wjVg/PLOSOne_formatting_sample_main_body.pdf> and 
<https://journals.plos.org/plosone/s/file?id=ba62/PLOSOne_formatting_sample_title_authors_affiliations.pdf>

**Reply:** Agreed. We have updated our manuscript according to the style requirements.

2. We note that the grant information you provided in the **‘Funding Information’ and ‘Financial Disclosure’** sections do not match.

When you resubmit, please ensure that you provide the correct grant numbers for the awards you received for your study in the ‘Funding Information’ section.

**Reply:** The research work was supported by a donation fund from Wu Jieh Yee Charitable Foundation Limited. The fund has no formal grant number.

3. We note that you have included the phrase “data not shown” in your manuscript. Unfortunately, this does not meet our data sharing requirements. PLOS does not permit references to inaccessible data. We require that authors **provide all relevant data within the paper, Supporting Information files, or in an acceptable, public repository**. Please add a citation to support this phrase or upload the data that corresponds with these findings to a stable repository (such as Figshare or Dryad) and provide and URLs, DOIs, or accession numbers that may be used to access these data. **Or, if the data are not a core part of the research being presented in your study, we ask that you remove the phrase that refers to these data**.

**Reply:** Agreed. This phrase was removed as the data are not a core part of the research.

4. Please review your reference list to ensure that it is complete and correct. If you have cited papers that have been retracted, please include the rationale for doing so in the manuscript text, or remove these references and replace them with relevant current references. **Any changes to the reference list should be mentioned in the rebuttal letter that accompanies your revised manuscript**. If you need to cite a retracted article, indicate the article’s retracted status in the References list and also include a citation and full reference for the retraction notice.

**Reply:**

The following references were newly added along with the amended manuscript:

38.	Geoffriau E, Denoue D, Rameau C. Assessment of genetic variation among asparagus (*Asparagus officinalis* L.) populations and cultivars: agromorphological and isozymic data. Euphytica. 1992; 61:169-179. doi:10.1007/BF00039655

39.	Mercati F, Riccardi P, Harkess A. Single nucleotide polymorphism-based parentage analysis and population structure in garden asparagus, a worldwide genetic stock classification. Mol Breeding. 2015. 35(59):1-12. doi:10.1007/s11032-015-0217-5

40.	Moreno R, Espejo JA, Cabrera A, Millán T, Gil J. Ploidic and Molecular Analysis of ‘Morado de Huetor’ asparagus (Asparagus officinalis L.) population; a Spanish tetraploid landrace. 2006; 53:729–736. Genet Resour Crop Evol. doi:10.1007/s10722-004-4717-0

82.	Obermeyer AA, Immelamn KL, Bos JJ. (1992). Asparagaceae. In: Leistner OA, du Plessis E, editors. Flora of Southern Africa. Volumn 5, Part 3. Dracaenaceae, Asparagaceae, Luzuriagaceae and Smilacaceae. Pretoria: National Botanical Institute; 1992. pp. 11-82.

93.	Lee YO, Kanno A, Kameya T. The physical map of the chloroplast DNA from Asparagus officinalis L. Theor Appl Genet. 1996;92:10-14.

94.	Kanno A, Lee YO, Kameya T. The structure of the chloroplast genome in members of the genus Asparagus. Theor Appl Genet. 1997;95:1196-1202.

95.	Sheng W, Chai X, Rao Y, Tu X & Du S. Complete chloroplast genome sequence of Asparagus (Asparagus officinalis L.) and its phylogenetic position within Asparagales. J Plant Breed Genet. 2017; 5(3):121-128.

96.	Li JR, Li SF, Wang J, Dong R, Zhu HW, Li N, et al. Characterization of the complete chloroplast genome of Asparagus setaceus. Mitochondrial DNA B Resour, 2019; 4(2):2639-2640. doi: 10.1080/23802359.2019.1643798

114.	Lee YO, Kanno A, Kameya T. Phylogenetic relationships in the genus *Asparagus* based on the restriction enzyme analysis of the chloroplast DNA. Japanese Journal of Breeding. 1997; 47:375-378.

The following reference was deleted according to the comment from Reviewer 1:

45.	Stajner N, Bohanec B, Javornik B. Genetic variability of economically important Asparagus species as revealed by genome size analysis and rDNA ITS polymorphisms. Plant Sci. 2002; 162(6):931-937. doi: 10.1016/S0168-9452(02)00039-0

Thank you very much for your kindly reviews and consideration.

Yours sincerely,

Dr. David TW LAU

Curator of the Shiu-ying Hu Herbarium

School of Life Sciences

The Chinese University of Hong Kong
