# Supplementary material for: Complete chloroplast genomes of Asparagus aethiopicus L., A. densiflorus (Kunth) Jessop ‘Myers’, and A. cochinchinensis (Lour.) Merr.: Comparative and phylogenetic analysis with congenerics
Source: PLoS One. 2022 Apr 25;17(4):e0266376. doi: 10.1371/journal.pone.0266376 (PMC9037925; doi:10.1371/journal.pone.0266376)
Supplement: Attachment — Submitted filename: Rebuttal Letter.docx [file pone.0266376.s007.docx]

Dear Dr. Šiler,

**Rebuttal Letter**

I am sincerely writing to you to response all your comments in the Decision Letter in 31^st^ January 2022. Our replies are listed as below.

**Original comments from Dr. Šiler:**

Following the rules of ICN (not ICNCP, since the studied taxa are not cultivated) is absolutely necessary in scientific literature and I fully support it. However, please bear in mind that abbreviating genus name does not oppose the ICN rules (the current version is Shenzhen Code, published by IAPT - https://www.iapt-taxon.org/nomen/main.php; for genera, please see https://www.iapt-taxon.org/nomen/pages/main/art_20.html). It is a common scientific practice to write a scientific name in full when it is first used or when several species from the same genus are being listed or discussed in the same paper or report. For subsequent uses, the genus can be abbreviated to its first letter followed by a period. You can abbreviate the genus name after its first use even when describing a different species within that genus, as long as there is no risk of confusing it for another genus or genera (which is not the case here, since only the genus *Asparagus* circulates throughout the manuscript). Therefore, by abbreviating the genus name (i) obeying the rules of ICN is not compromised, ii) nomenclatural treatment is presented accurately (no confusion to other genera starting with "*A*."), and iii) if a species is described in a source article as e.g., "*Asparagus aethiopicus*", I do not see the reason why in consequent articles dealing with the same species it wouldn't be abbreviated as "*A. aethiopicus*" if the genus name was already mentioned earlier in the text. The same applies to the main title: reprising the genus name three times is space-consuming and quite exhausting for reading. I suggest abbreviating here the genus name for the second and the third species as well (also, replace "&" with "and" here and elsewhere in the text). The exceptions might be L178-179 (cites the original text), L197-200 (cites the original text), L218-220 (only for mentioning *Asparagus myersii*, since a binomial name has been announced), L222-223 (cites the original text), Table 1, L614-616 (a full name has been announced), L647-648 (as it complies with the ICN rules), L649-651 (but being the common name, "asparagus” should stand in lowercase).

Moreover, **vernacular expressions** such as L101 (circumscription of *Asparagus*), L117 (relationships within *Asparagus*), L125 (only one native *Asparagus*), L126 (Other common exotic *Asparagus*), L232 (7 *Asparagus* were examined) (and in many other places) were not clarified as required in the previous review round.

102-107: The terms **"genus" and "subgenus" should stand in lowercase**.

L127-129 and elsewhere in the text: I must repeat: **common names should be written in lowercase**, i.e., "sprengeri asparagus" (however, I'm not aware of this Latinized common name, but "Sprenger's asparagus" might be acceptable, after Carl Ludwig Sprenger), "lace fern", etc.

The authors are urged to meticulously check the text once again for proper English usage while following the comments stated above. I still strongly encourage the authors to have the MS proofread by a native English speaker or professional editing agency.

**Reply:** Thank you so much for your detailed clarification. Please see the reply of the subtracted comments from your comments:

**Comment 1:** Therefore, by abbreviating the genus name (i) obeying the rules of ICN is not compromised, ii) nomenclatural treatment is presented accurately (no confusion to other genera starting with "*A*."), and iii) if a species is described in a source article as e.g., "*Asparagus aethiopicus*", I do not see the reason why in consequent articles dealing with the same species it wouldn't be abbreviated as "*A. aethiopicus*" if the genus name was already mentioned earlier in the text.

**Reply:** Agreed. Please see the amendment in the manuscript.

**Comment 2:** The same applies to the main title: reprising the genus name three times is space-consuming and quite exhausting for reading. I suggest abbreviating here the genus name for the second and the third species as well …

**Reply:** Agreed. Please see the amendment in the manuscript.

**Comment 3:** (also, replace "&" with "and" here and elsewhere in the text)

**Reply:** Agreed. All the sign “&” were placed by the word “and” in the main text and title.

**Comment 4:** The exceptions might be L178-179 (cites the original text), L197-200 (cites the original text), L218-220 (only for mentioning *Asparagus myersii*, since a binomial name has been announced), L222-223 (cites the original text), Table 1, L614-616 (a full name has been announced), L647-648 (as it complies with the ICN rules), L649-651 (but being the common name, "asparagus” should stand in lowercase).

**Reply:** Thank you so much for your consideration for these exception. However, two more exceptions we would like to request:

L165-167: In 1890, Regel published the name ***Asparagus sprengeri*** based on the cultivated plants growing in Natal.

Reason: Cites the original text

L187-188: Both names ***Aspararagopsis aethiopica*** (and later, ***Asparagus aethiopicus***) and ***Asparagopsis densiflora*** were adopted in parallel for 116 years, from 1850 to 1965.

Reason: Please noticed that there are two genera involved: ***Asparagopsis*** and ***Asparagus***. Both the abbreviation of these two genera are “*A.*” which would be confusing. Moreover, please noticed that *Asparagopsis aethiopica* and *Asparagopsis densiflora* were the name published by Kunth in 1850 (Please refer to the S2 Table).

**Comment 5:** Moreover, **vernacular expressions** such as L101 (circumscription of *Asparagus*), L117 (relationships within *Asparagus*), L125 (only one native *Asparagus*), L126 (Other common exotic *Asparagus*), L232 (7 *Asparagus* were examined) (and in many other places) were not clarified as required in the previous review round.

**Reply:** Agreed. Please see the amendment in the manuscript.

**Comment 6:** 102-107: The terms **"genus" and "subgenus" should stand in lowercase**.

**Reply:** Agreed. Please see the amendment in the manuscript.

**Comment 7:** L127-129 and elsewhere in the text: I must repeat: **common names should be written in lowercase**, i.e., "sprengeri asparagus" (however, I'm not aware of this Latinized common name, but "Sprenger's asparagus" might be acceptable, after Carl Ludwig Sprenger), "lace fern", etc.

**Reply:** Agreed. The common name “Sprenger’s asparagus” is adopted.

**Comment 8:** The authors are urged to meticulously check the text once again for proper English usage while following the comments stated above. I still strongly encourage the authors to have the MS proofread by a native English speaker or professional editing agency.

**Reply:** Agreed. We employed a professional editing agency to proofread the manuscript.

Thank you very much for your kindly reviews and consideration.

Yours sincerely,

Dr. David TW LAU

Curator of the Shiu-ying Hu Herbarium

School of Life Sciences

The Chinese University of Hong Kong
